# Synthesis and crystal structure of a new tetra­nuclear copper(II) complex based on the Schiff base (*E*)-2-[(2-hy­droxy-5-meth­oxy­benzyl­idene)amino]­benzoic acid

**DOI:** 10.1107/S2056989026005554

**Published:** 2026-06-05

**Authors:** Dawei Li, Wanying Qi, Jingjing Wang, Haiyang Dong, Kuilin Lv, Kaige Shi

**Affiliations:** a School of Chemistry and Chemical Engineering Henan Engineering Technology Research Center for Green Catalytic and Atom Economic Conversion of Coal-based Benzene Zhengzhou Normal University,Zhengzhou 450044 Henan Province People’s Republic of China; Institute of Chemistry, Chinese Academy of Sciences

**Keywords:** crystal structure, tetra­nuclear copper complex, Schiff base ligand

## Abstract

The title complex, bis­{μ_4_-(*E*)-2-[(5-meth­oxy-2-oxido­benzyl­idene)amino]­benzoato}bis­{μ_2_-(*E*)-2-[(5-meth­oxy-2-oxido­benzyl­idene)amino]­benzoato}tetra­copper(II), [Cu_4_(C_15_H_11_NO_4_)_4_] or Cu_4_(*L*)_4_, was synthesized by the solvothermal reaction of (*E*)-2-[(2-hy­droxy-5-meth­oxy­benzyl­idene)amino]­benzoic acid (H_2_*L*) with copper(II) chloride. It crystallizes in the triclinic system with space group *P*ī. The tetra­nuclear structure consists of two fully symmetric dinuclear copper moieties. In the dinuclear copper structural unit, both copper(II) metal centers exhibit a five-coordinate NO_4_ environment. Of the four coordinating oxygen atoms, two are phenolate oxygen atoms acting as bridges from two different ligands, while the other two oxygen atoms are derived from the carboxyl groups of two distinct ligands, respectively. *SHAPE* analysis indicates that both copper(II) centers adopt a distorted trigonal–bipyramidal (*D*_3*h*_) geometry. C—H⋯O hydrogen bonds and C—H⋯π inter­actions contribute to the cohesion of the crystal packing.

## Abstract 2

A novel tetra­nuclear Cu^2+^ complex was solvothermally constructed from a salicyl­aldehyde-based Schiff base ligand, which possesses a centrosymmetric framework with two dinuclear Cu^2+^ units; all Cu^2+^ centers adopt a distorted trigonal–bipyramidal coordination geometry. C—H⋯O hydrogen bonds and C—H⋯π inter­actions further consolidate the packing.

## 1. Chemical context

Coordination polymers constructed from Schiff base ligands bearing phenolic hydroxyl and carboxyl groups have attracted extensive research inter­est, owing to their elegant structural topologies and fascinating magnetic properties (Allendorf *et al.*, 2009 ▸; Karahan *et al.*, 2015 ▸). Such ligands are distinguished by facile synthesis, flexible structural modification, and strong coordination capability (Zhang *et al.*, 2012 ▸). In particular, the corresponding metal complexes are easily accessible, feature diversifiable and tunable structures, and possess desirable magnetic behaviors as well as biological activities (Karahan *et al.*, 2015 ▸). The rational design of building blocks, together with the utilization of coordination bonds and non-covalent inter­actions to self-assemble multidimensional supra­molecular aggregates with delicate architectures for potential functional material applications, represents a vital research hotspot in supra­molecular chemistry and crystal engineering (Sasmal *et al.*, 2011 ▸). In light of the above, this paper reports the synthesis and crystal structure of the title complex.
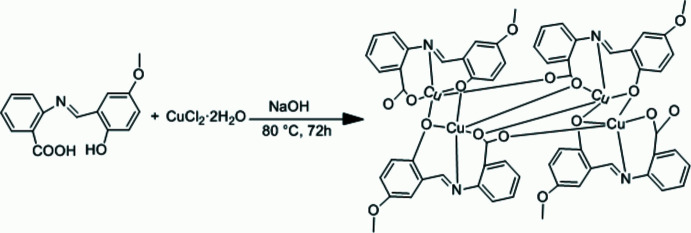


## 2. Structural commentary

**Figure 1:**
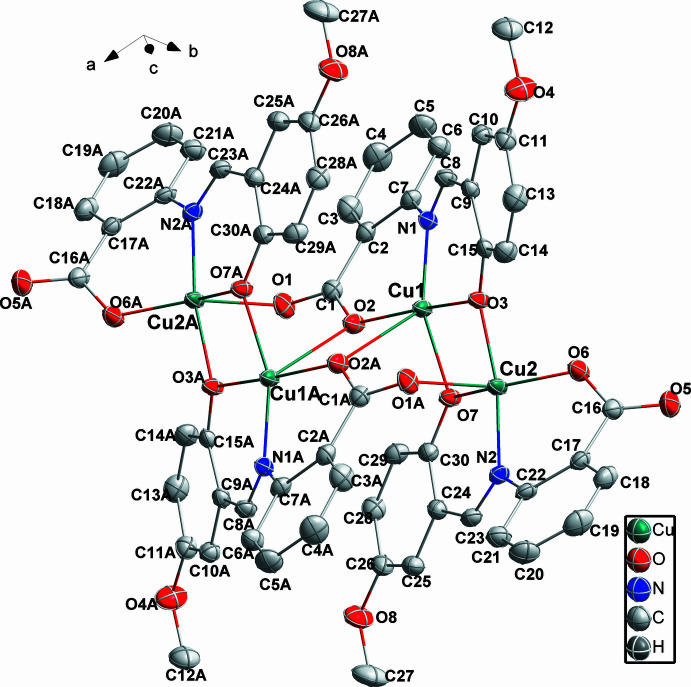
Mol­ecular structure of the title compound with 50% probability ellipsoids. For clarity, H atoms are not shown.

**Figure 2:**
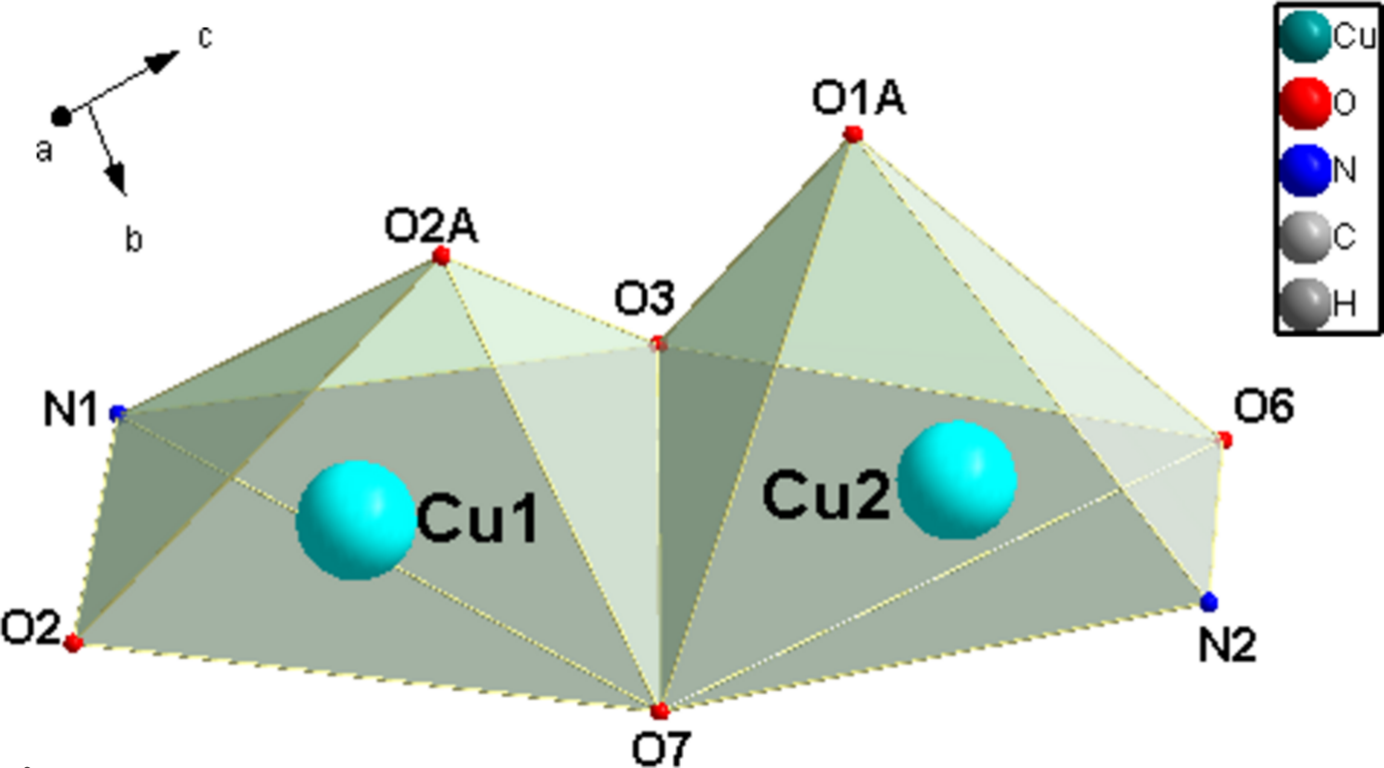
Coordination polyhedra of Cu^II^ ions. Colour: cyan (Cu), red (O), blue (N).

**Figure 3:**
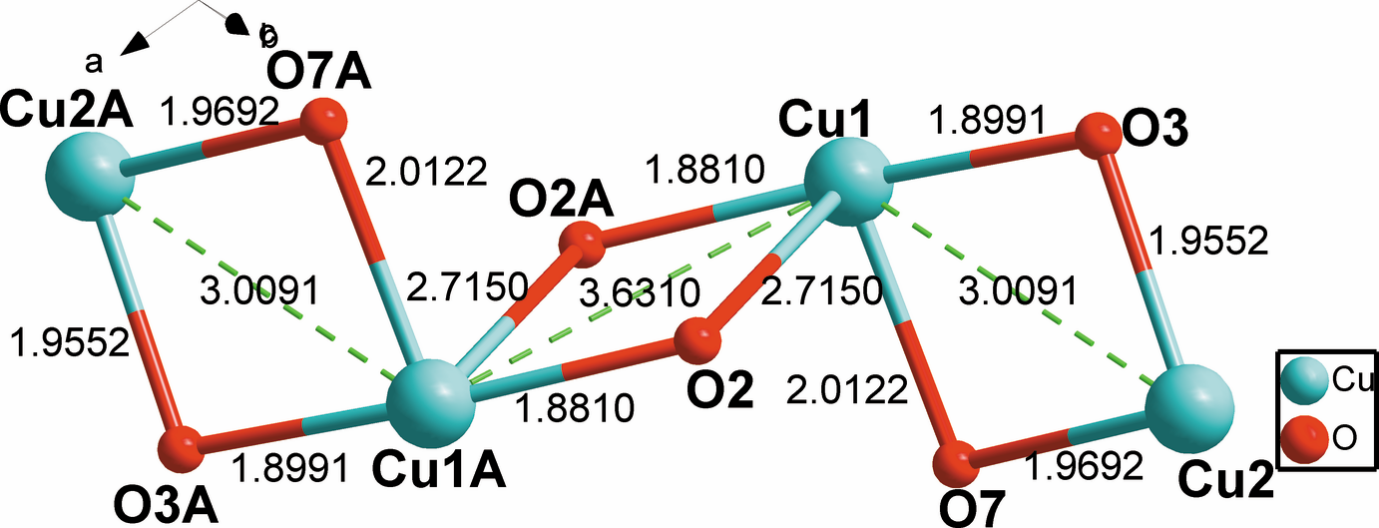
Zigzag chain configuration in the complex.

The title Cu_4_(*L*)_4_ complex crystallizes in the triclinic crystal system in space group *P*ī. The tetra­nuclear structural motif is constructed from two symmetry-equivalent dinuclear Cu^II^ subunits bridged by phenolic hydroxyl and carboxyl­ate groups (Fig. 1 ▸). In the dinuclear Cu^II^ unit, each central divalent copper ion adopts a five-coordinate configuration and displays a distorted trigonal–bipyramidal (*D*_3*h*_) geometry (Fig. 2 ▸). Within the coordination polyhedron of each Cu^II^ center, the coordinating atoms consist of one nitro­gen atom (N1), one carboxyl­ate oxygen atom (O2) and one phenolate oxygen atom (O3) all originating from a single ligand, one carboxyl­ate oxygen atom (O2*A*) from a second ligand, and one phenolate oxygen atom (O7) from a third ligand. In general, five-coordinate Cu^II^ ions are typically tend to adopt square-pyramidal coordination geometries. The distinctive distorted trigonal–bipyramidal coordination environment of the Cu^II^ atom in the title complex is mainly induced by the inherent steric hindrance of the ligand framework, and intra­molecular hydrogen-bonding inter­actions (Table 1 ▸) further contribute to the structural stabilization. The two dinuclear Cu^II^ cores form an approximately square-planar arrangement. Two such nearly square dinuclear moieties are further inter­connected *via* two carboxyl­ate oxygen bridges, affording a zigzag chain-shaped three-fused cyclic architecture (Fig. 3 ▸). For the four Cu^II^ centers, the adjacent Cu⋯Cu inter­atomic distances are 3.0091 (3), 3.6310 (4) and 3.0091 (3) Å. The Cu—N bond lengths are 1.9530 (14) and 1.9295 (15) Å, while the Cu—O bond distances are in the range 1.8810 (12) to 2.3276 (14) Å. A continuous shape analysis of the coordination geometries for the two Cu^II^ centers within the dinuclear fragment was performed by means of the *SHAPE 2.0* program (Llunell *et al.*, 2013 ▸), and the corresponding qu­anti­tative parameters are summarized in Table 2 ▸.

## 3. Supra­molecular features

**Figure 4:**
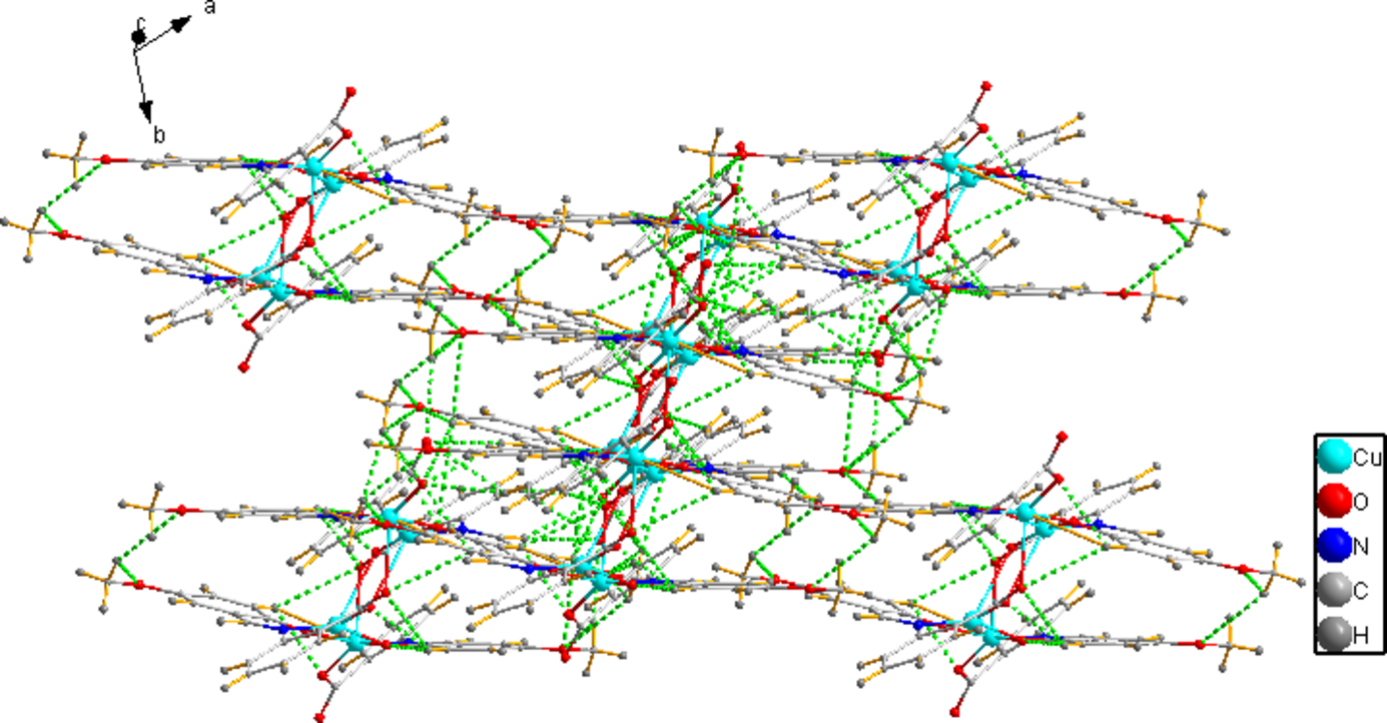
The crystal packing with the C—H⋯O hydrogen bonds shown as green dashed lines.

In the crystal, weak C—H⋯O hydrogen bonds and C—H⋯π inter­actions inter­connect the complex mol­ecules to construct a three-dimensional supra­molecular network (Fig. 4 ▸, Table 1 ▸).

## 4. Database survey

A search was performed using the Cambridge Structural Database (CSD, Version 5.37, Update 1; Groom *et al.*, 2016 ▸) to retrieve linear tetra­nuclear copper(II) complexes constructed from Schiff base ligands structurally analogous to the title compound. Only few related crystal structures were identified: the complex [Cu(salpd-*μ*-O,O′)(μ-*L*)Cu(*μ*-CH_3_O)_2_Cu(*μ*-*L*)salpd-*μ*-O,*O*′)Cu], (*L* = acetate or formate ions) (KEPZAG; Fukuhara *et al.*, 1989 ▸); a series of linear tetra­nuclear copper(II) complexes [Cu_4_(bzacpro)_2_(C_2_H_5_O)_2_], [Cu_4_(bzacbu)_2_(CH_3_O)_2_], [Cu_4_(bzacpen)_2_(CH_3_CO_2_)_2_], and [Cu_4_(bzacpen)_2_O]·H_2_O·(*N*,*N*′-bis­(1-methyl-3-hy­droxy-3-phen­yl-2-propen-1-yl­idene)-1,3-di­amino-2-propanol (H_3_bzacpro), *N*,*N*′-bis­(1-methyl-3-hy­droxy-3-phenyl-2-propen-1-yl­idene)-1,4-di­amino-2-butanol (H_3_bzacbu), and *N*,*N*′-bis­(1-methyl-3-hy­droxy-3-phenyl-2-propen-1-yl­idene)-1,5-di­amino-3-penta­nol (H_3_bzacpen) (EHUPEC, EHUPOM, EHUPUS and EHUQAZ; Mikuriya *et al.*, 2002 ▸); the complex [Cu_4_(2,2′-bpy)_6_(ip)_2_(H_2_O)_2_]·4ClO_4_·6H_2_O (2,2′-bpy = 2,2′-bi­pyridine and H_2_ip = isophthalic acid) (CCDC 661868; Zhang *et al.*, 2011 ▸). Among the six linear tetra­nuclear copper(II) aggregates reported in their work, the Cu—O and Cu—N bond lengths at each coordination site are well consistent with those of the title compound in this study. In addition, two linear tetra­nuclear copper(II) complexes, formulated as [Cu_4_(*L*_1_)_2_(μ-*N*_3_)_2_(*N*_3_)_2_] (**1**) and [Cu_4_(*L*_2_)_2_(μ-*N*_3_)_2_(*N*_3_)_2_] (**2**). [*L*_1_= *N*,*N*′-bis­(salicyl­idene)di­amino­propane (salpn) and *L*_2_=*N*,*N*′-bis­(salicyl­idene)di­amino­benzene ­(salophen)] (AGEZAQ and AGEZEU; Pandey *et al.*, 2018 ▸). These two linear tetra­nuclear copper(II) species share a fundamental structural framework identical to that of the title compound.

## 5. Synthesis and crystallization

A mixture of CuCl_2_·2H_2_O (0.05 mmol), the ligand H_2_*L* (0.05 mmol) and NaOH (0.1 mmol) was placed into a Pyrex tube (about 12 mL) together with ethanol (2 mL) and deionized water (2 mL). The sealed tube was heated at 353 K under autogenous pressure for 72 h. Dark-green elongated crystals suitable for single-crystal X-ray diffraction analysis were successfully obtained. Based on copper, the yield of the title complex was calculated to be 56% (0.009 g).

## 6. Refinement

Crystal data, data collection and structure refinement details are summarized in Table 3 ▸. All C—H hydrogen atoms were generated at idealized geometrical positions, with methyl hydrogen atoms allowed to rotate while remaining non-tilting. These hydrogen atoms were refined isotropically under the thermal constraint: *U*_iso_(H)=1.2*U*_eq_(C) (1.5*U*_eq_(C) for methyl hydrogen atoms).

## Supplementary Material

Crystal structure: contains datablock(s) I. DOI: 10.1107/S2056989026005554/nx2036sup1.cif

Structure factors: contains datablock(s) I. DOI: 10.1107/S2056989026005554/nx2036Isup2.hkl

CCDC reference: 2544733

Additional supporting information:  crystallographic information; 3D view; checkCIF report

## Figures and Tables

**Table 1:** Hydrogen-bond geometry (Å, °)

*D*—H⋯*A*	*D*—H	H⋯*A*	*D*⋯*A*	*D*—H⋯*A*
C14—H14⋯O6	0.93	2.55	3.112 (3)	119
C29—H29⋯O2	0.93	2.28	2.945 (2)	128
C23—H23⋯O5^i^	0.93	2.58	3.432 (2)	153
C27—H27*A*⋯O5^ii^	0.96	2.63	3.567 (3)	165

Symmetry codes: (i) 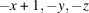; (ii) 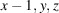.

**Table 2:** Agreement factor between the coordination polyhedron of the Cu^II^ ion in complex **1** and the various ideal polyhedra calculated by the *SHAPE* program

Atom	PP-5 (*D*_5*h*_)	vOC-5 (*C*_4*v*_)	TBPY-5 (*D*_3*h*_)	SPY-5 (*C*_4*v*_)	JTBPY-5 (*D*_3*h*_)
Cu1	21.750	7.133	6.260	6.286	10.635
Cu2	29.860	4.817	1.702	3.670	5.327

PP-5 (*D*_5*h*_): penta­gon; vOC-5 (*C*_4*v*_) vacant octa­hedron; TBPY-5 (*D*_3*h*_): trigonal bipyramid; SPY-5 (*C*_4*v*_): spherical square pyramid; JTBPY-5 (*D*_3*h*_): Johnson trigonal bipyramid J12.

**Table 3:** Experimental details

Crystal data	
Chemical formula	[Cu_4_(C_15_H_11_NO_4_)_4_]
*M*_r_	1331.15
Crystal system, space group	Triclinic, *P* 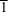
Temperature (K)	296
*a*, *b*, *c* (Å)	10.7949 (6), 11.0214 (6), 11.9680 (6)
α, β, γ (°)	103.651 (2), 95.318 (2), 109.881 (2)
*V* (Å^3^)	1277.69 (12)
*Z*	1
Radiation type	Mo *K*α
μ (mm^−1^)	1.73
Crystal size (mm)	0.44 × 0.42 × 0.26

Data collection	
Diffractometer	Bruker CCD area detector
Absorption correction	Multi-scan (*SADABS*; Krause *et al.*, 2015 ▸)
*T*_min_, *T*_max_	0.546, 0.746
No. of measured, independent and observed [*I* > 2σ(*I*)] reflections	48410, 5891, 5107
*R*_int_	0.049
(sin θ/λ)_max_ (Å^−1^)	0.651

Refinement	
*R*[*F*^2^ > 2σ(*F*^2^)], *wR*(*F*^2^), *S*	0.026, 0.069, 1.03
No. of reflections	5891
No. of parameters	381
H-atom treatment	H-atom parameters constrained
Δρ_max_, Δρ_min_ (e Å^−3^)	0.33, −0.44

Computer programs: *APEX2* and *SAINT* (Bruker, 2014 ▸), *SHELXT* (Sheldrick, 2015*a* ▸), *SHELXL* (Sheldrick, 2015*b* ▸) and *OLEX2* (Dolomanov *et al.*, 2009 ▸).

## supplementary crystallographic information

### Bis{µ4-(E)-2-[(5-methoxy-2-oxidobenzylidene)amino]benzoato}bis{µ2-(E)-2-[(5-methoxy-2-oxidobenzylidene)amino]benzoato}tetracopper(II) Crystal data

**Table:**

[Cu_4_(C_15_H_11_NO_4_)_4_]	*Z* = 1
*M**_r_* = 1331.15	*F*(000) = 676
Triclinic, *P*1	*D*_x_ = 1.730 Mg m^−^^3^
*a* = 10.7949 (6) Å	Mo *K*α radiation, λ = 0.71073 Å
*b* = 11.0214 (6) Å	Cell parameters from 9991 reflections
*c* = 11.9680 (6) Å	θ = 2.4–27.5°
α = 103.651 (2)°	µ = 1.73 mm^−^^1^
β = 95.318 (2)°	*T* = 296 K
γ = 109.881 (2)°	Block, dull greenish blue
*V* = 1277.69 (12) Å^3^	0.44 × 0.42 × 0.26 mm

### Bis{µ4-(E)-2-[(5-methoxy-2-oxidobenzylidene)amino]benzoato}bis{µ2-(E)-2-[(5-methoxy-2-oxidobenzylidene)amino]benzoato}tetracopper(II) Data collection

**Table:**

Bruker CCD area detector diffractometer	5107 reflections with *I* > 2σ(*I*)
Graphite monochromator	*R*_int_ = 0.049
phi and ω scans	θ_max_ = 27.6°, θ_min_ = 2.4°
Absorption correction: multi-scan (SADABS; Krause *et al.*, 2015)	*h* = −14→14
*T*_min_ = 0.546, *T*_max_ = 0.746	*k* = −14→14
48410 measured reflections	*l* = −15→15
5891 independent reflections	

### Bis{µ4-(E)-2-[(5-methoxy-2-oxidobenzylidene)amino]benzoato}bis{µ2-(E)-2-[(5-methoxy-2-oxidobenzylidene)amino]benzoato}tetracopper(II) Refinement

**Table:**

Refinement on *F*^2^	Primary atom site location: dual
Least-squares matrix: full	Hydrogen site location: inferred from neighbouring sites
*R*[*F*^2^ > 2σ(*F*^2^)] = 0.026	H-atom parameters constrained
*wR*(*F*^2^) = 0.069	*w* = 1/[σ^2^(*F*_o_^2^) + (0.0282*P*)^2^ + 0.9769*P*] where *P* = (*F*_o_^2^ + 2*F*_c_^2^)/3
*S* = 1.03	(Δ/σ)_max_ = 0.002
5891 reflections	Δρ_max_ = 0.33 e Å^−^^3^
381 parameters	Δρ_min_ = −0.44 e Å^−^^3^
0 restraints	

### Bis{µ4-(E)-2-[(5-methoxy-2-oxidobenzylidene)amino]benzoato}bis{µ2-(E)-2-[(5-methoxy-2-oxidobenzylidene)amino]benzoato}tetracopper(II) Special details

**Table:**

Geometry. All esds (except the esd in the dihedral angle between two l.s. planes) are estimated using the full covariance matrix. The cell esds are taken into account individually in the estimation of esds in distances, angles and torsion angles; correlations between esds in cell parameters are only used when they are defined by crystal symmetry. An approximate (isotropic) treatment of cell esds is used for estimating esds involving l.s. planes.

### Bis{µ4-(E)-2-[(5-methoxy-2-oxidobenzylidene)amino]benzoato}bis{µ2-(E)-2-[(5-methoxy-2-oxidobenzylidene)amino]benzoato}tetracopper(II) Fractional atomic coordinates and isotropic or equivalent isotropic displacement parameters (Å^2^)

**Table:**

	*x*	*y*	*z*	*U*_iso_*/*U*_eq_	
Cu1	0.64016 (2)	0.44708 (2)	0.52175 (2)	0.01786 (6)	
Cu2	0.63788 (2)	0.31527 (2)	0.27047 (2)	0.01985 (7)	
O7	0.52279 (12)	0.28733 (12)	0.38781 (10)	0.0202 (3)	
O3	0.75631 (12)	0.45024 (13)	0.41168 (10)	0.0196 (3)	
O2	0.50759 (12)	0.46228 (13)	0.60953 (11)	0.0227 (3)	
O1	0.41991 (13)	0.51054 (14)	0.76564 (11)	0.0254 (3)	
O6	0.77644 (13)	0.26899 (15)	0.20522 (11)	0.0290 (3)	
O4	1.24853 (15)	0.84619 (15)	0.44292 (13)	0.0358 (4)	
O5	0.84082 (15)	0.13655 (16)	0.07352 (13)	0.0369 (4)	
N2	0.49854 (15)	0.19756 (15)	0.13786 (13)	0.0197 (3)	
N1	0.78775 (14)	0.55439 (15)	0.65415 (12)	0.0170 (3)	
O8	−0.01846 (14)	−0.02347 (15)	0.31525 (14)	0.0353 (3)	
C1	0.51603 (18)	0.49910 (17)	0.72134 (16)	0.0197 (4)	
C15	0.87163 (17)	0.55335 (17)	0.42150 (15)	0.0174 (3)	
C7	0.77072 (18)	0.55219 (17)	0.77071 (15)	0.0184 (3)	
C30	0.39099 (17)	0.21157 (17)	0.36530 (16)	0.0192 (3)	
C10	1.07282 (18)	0.73894 (18)	0.54070 (16)	0.0210 (4)	
H10	1.1224	0.7938	0.6141	0.025*	
C23	0.37476 (18)	0.13821 (18)	0.14529 (16)	0.0211 (4)	
H23	0.3153	0.0877	0.0750	0.025*	
C2	0.64427 (18)	0.52756 (17)	0.80196 (15)	0.0191 (3)	
C29	0.32105 (19)	0.19819 (19)	0.45757 (16)	0.0236 (4)	
H29	0.3668	0.2427	0.5342	0.028*	
C8	0.90317 (18)	0.63124 (18)	0.64082 (15)	0.0191 (3)	
H8	0.9647	0.6868	0.7087	0.023*	
C16	0.76517 (18)	0.19539 (19)	0.10052 (17)	0.0240 (4)	
C22	0.53234 (18)	0.19068 (17)	0.02414 (15)	0.0208 (4)	
C6	0.88014 (19)	0.5737 (2)	0.85524 (16)	0.0243 (4)	
H6	0.9639	0.5880	0.8347	0.029*	
C9	0.94566 (17)	0.63919 (18)	0.53175 (15)	0.0174 (3)	
C24	0.31906 (18)	0.14162 (18)	0.24996 (16)	0.0202 (4)	
C11	1.12378 (19)	0.75570 (19)	0.44233 (17)	0.0232 (4)	
C26	0.11468 (18)	0.05233 (19)	0.32383 (18)	0.0246 (4)	
C14	0.92307 (19)	0.5770 (2)	0.32232 (16)	0.0236 (4)	
H14	0.8734	0.5249	0.2483	0.028*	
C13	1.0464 (2)	0.6763 (2)	0.33279 (17)	0.0271 (4)	
H13	1.0785	0.6905	0.2656	0.033*	
C28	0.18627 (19)	0.12073 (19)	0.43753 (17)	0.0259 (4)	
H28	0.1426	0.1140	0.5005	0.031*	
C17	0.65857 (18)	0.18987 (18)	0.00625 (16)	0.0216 (4)	
C3	0.6339 (2)	0.5301 (2)	0.91729 (17)	0.0281 (4)	
H3	0.5506	0.5163	0.9388	0.034*	
C18	0.6888 (2)	0.1836 (2)	−0.10535 (17)	0.0283 (4)	
H18	0.7713	0.1803	−0.1186	0.034*	
C21	0.4427 (2)	0.1897 (2)	−0.06769 (17)	0.0271 (4)	
H21	0.3595	0.1919	−0.0555	0.032*	
C19	0.5997 (2)	0.1820 (2)	−0.19642 (18)	0.0338 (5)	
H19	0.6223	0.1786	−0.2699	0.041*	
C5	0.8660 (2)	0.5741 (2)	0.96909 (17)	0.0297 (4)	
H5	0.9400	0.5890	1.0243	0.036*	
C25	0.17955 (18)	0.06293 (19)	0.23137 (17)	0.0241 (4)	
H25	0.1318	0.0181	0.1554	0.029*	
C20	0.4764 (2)	0.1855 (2)	−0.17737 (18)	0.0325 (5)	
H20	0.4159	0.1851	−0.2382	0.039*	
C4	0.7419 (2)	0.5523 (2)	1.00098 (17)	0.0324 (5)	
H4	0.7317	0.5527	1.0774	0.039*	
C12	1.3242 (2)	0.9314 (2)	0.55455 (19)	0.0342 (5)	
H12A	1.4053	0.9963	0.5449	0.051*	
H12B	1.3463	0.8783	0.6003	0.051*	
H12C	1.2723	0.9773	0.5939	0.051*	
C27	−0.0917 (2)	−0.1014 (3)	0.2011 (2)	0.0463 (6)	
H27A	−0.1006	−0.0426	0.1558	0.069*	
H27B	−0.1792	−0.1592	0.2063	0.069*	
H27C	−0.0450	−0.1551	0.1640	0.069*	

### Bis{µ4-(E)-2-[(5-methoxy-2-oxidobenzylidene)amino]benzoato}bis{µ2-(E)-2-[(5-methoxy-2-oxidobenzylidene)amino]benzoato}tetracopper(II) Atomic displacement parameters (Å^2^)

**Table:**

	*U*^11^	*U*^22^	*U*^33^	*U*^12^	*U*^13^	*U*^23^
Cu1	0.01381 (11)	0.02307 (12)	0.01270 (11)	0.00514 (9)	0.00312 (8)	0.00024 (8)
Cu2	0.01423 (11)	0.02633 (12)	0.01317 (11)	0.00607 (9)	0.00153 (8)	−0.00229 (9)
O7	0.0166 (6)	0.0229 (6)	0.0155 (6)	0.0037 (5)	0.0029 (5)	0.0009 (5)
O3	0.0139 (6)	0.0260 (6)	0.0134 (6)	0.0045 (5)	0.0025 (5)	0.0001 (5)
O2	0.0180 (6)	0.0296 (7)	0.0161 (6)	0.0071 (5)	0.0052 (5)	0.0003 (5)
O1	0.0226 (7)	0.0365 (8)	0.0217 (7)	0.0141 (6)	0.0114 (5)	0.0092 (6)
O6	0.0197 (7)	0.0425 (8)	0.0194 (7)	0.0144 (6)	0.0016 (5)	−0.0042 (6)
O4	0.0296 (8)	0.0351 (8)	0.0319 (8)	−0.0017 (6)	0.0149 (6)	0.0067 (6)
O5	0.0294 (8)	0.0448 (9)	0.0349 (8)	0.0219 (7)	0.0051 (6)	−0.0033 (7)
N2	0.0190 (7)	0.0212 (7)	0.0149 (7)	0.0063 (6)	0.0028 (6)	−0.0002 (6)
N1	0.0149 (7)	0.0222 (7)	0.0131 (7)	0.0066 (6)	0.0038 (5)	0.0034 (6)
O8	0.0180 (7)	0.0395 (8)	0.0386 (9)	0.0010 (6)	0.0072 (6)	0.0067 (7)
C1	0.0202 (9)	0.0187 (8)	0.0200 (9)	0.0060 (7)	0.0072 (7)	0.0059 (7)
C15	0.0157 (8)	0.0216 (8)	0.0175 (8)	0.0106 (7)	0.0032 (6)	0.0047 (7)
C7	0.0219 (9)	0.0200 (8)	0.0126 (8)	0.0074 (7)	0.0049 (7)	0.0036 (7)
C30	0.0178 (8)	0.0172 (8)	0.0216 (9)	0.0062 (7)	0.0035 (7)	0.0042 (7)
C10	0.0187 (9)	0.0214 (9)	0.0201 (9)	0.0058 (7)	0.0043 (7)	0.0031 (7)
C23	0.0189 (9)	0.0208 (9)	0.0179 (8)	0.0056 (7)	0.0000 (7)	−0.0007 (7)
C2	0.0218 (9)	0.0190 (8)	0.0165 (8)	0.0077 (7)	0.0065 (7)	0.0040 (7)
C29	0.0255 (10)	0.0226 (9)	0.0192 (9)	0.0052 (8)	0.0051 (7)	0.0048 (7)
C8	0.0176 (8)	0.0226 (9)	0.0140 (8)	0.0061 (7)	0.0013 (7)	0.0025 (7)
C16	0.0183 (9)	0.0251 (9)	0.0230 (9)	0.0055 (7)	0.0058 (7)	−0.0001 (7)
C22	0.0206 (9)	0.0181 (8)	0.0157 (9)	0.0029 (7)	0.0024 (7)	−0.0031 (7)
C6	0.0212 (9)	0.0324 (10)	0.0181 (9)	0.0099 (8)	0.0044 (7)	0.0052 (8)
C9	0.0161 (8)	0.0218 (8)	0.0162 (8)	0.0093 (7)	0.0043 (6)	0.0053 (7)
C24	0.0176 (9)	0.0197 (8)	0.0210 (9)	0.0059 (7)	0.0037 (7)	0.0035 (7)
C11	0.0216 (9)	0.0228 (9)	0.0261 (10)	0.0073 (7)	0.0098 (8)	0.0083 (8)
C26	0.0178 (9)	0.0227 (9)	0.0314 (10)	0.0057 (7)	0.0056 (8)	0.0069 (8)
C14	0.0253 (10)	0.0307 (10)	0.0143 (9)	0.0107 (8)	0.0048 (7)	0.0045 (7)
C13	0.0328 (11)	0.0316 (10)	0.0207 (10)	0.0122 (9)	0.0127 (8)	0.0110 (8)
C28	0.0248 (10)	0.0251 (9)	0.0274 (10)	0.0068 (8)	0.0105 (8)	0.0085 (8)
C17	0.0224 (9)	0.0174 (8)	0.0195 (9)	0.0053 (7)	0.0044 (7)	−0.0016 (7)
C3	0.0291 (10)	0.0374 (11)	0.0221 (10)	0.0130 (9)	0.0128 (8)	0.0123 (8)
C18	0.0281 (10)	0.0267 (10)	0.0251 (10)	0.0077 (8)	0.0103 (8)	−0.0001 (8)
C21	0.0230 (10)	0.0304 (10)	0.0217 (10)	0.0070 (8)	0.0018 (8)	0.0017 (8)
C19	0.0416 (12)	0.0362 (11)	0.0183 (10)	0.0096 (10)	0.0117 (9)	0.0030 (8)
C5	0.0322 (11)	0.0394 (12)	0.0171 (9)	0.0145 (9)	0.0007 (8)	0.0072 (8)
C25	0.0185 (9)	0.0235 (9)	0.0248 (10)	0.0052 (7)	0.0010 (7)	0.0019 (7)
C20	0.0346 (11)	0.0380 (12)	0.0184 (10)	0.0088 (9)	0.0008 (8)	0.0044 (8)
C4	0.0405 (12)	0.0456 (12)	0.0158 (9)	0.0183 (10)	0.0097 (8)	0.0121 (9)
C12	0.0241 (10)	0.0318 (11)	0.0386 (12)	0.0005 (9)	0.0065 (9)	0.0102 (9)
C27	0.0199 (11)	0.0551 (15)	0.0461 (14)	−0.0022 (10)	0.0002 (10)	0.0085 (12)

### Bis{µ4-(E)-2-[(5-methoxy-2-oxidobenzylidene)amino]benzoato}bis{µ2-(E)-2-[(5-methoxy-2-oxidobenzylidene)amino]benzoato}tetracopper(II) Geometric parameters (Å, º)

**Table:**

Cu1—Cu2	3.0091 (3)	C29—H29	0.9300
Cu1—O7	2.0122 (12)	C29—C28	1.377 (3)
Cu1—O3	1.8991 (12)	C8—H8	0.9300
Cu1—O2	1.8810 (12)	C8—C9	1.435 (2)
Cu1—N1	1.9530 (14)	C16—C17	1.510 (3)
Cu2—O7	1.9692 (12)	C22—C17	1.401 (3)
Cu2—O3	1.9552 (12)	C22—C21	1.391 (3)
Cu2—O1^i^	2.3276 (14)	C6—H6	0.9300
Cu2—O6	1.9117 (13)	C6—C5	1.384 (3)
Cu2—N2	1.9295 (15)	C24—C25	1.424 (3)
O7—C30	1.345 (2)	C11—C13	1.393 (3)
O3—C15	1.343 (2)	C26—C28	1.394 (3)
O2—C1	1.288 (2)	C26—C25	1.370 (3)
O1—Cu2^i^	2.3276 (14)	C14—H14	0.9300
O1—C1	1.238 (2)	C14—C13	1.378 (3)
O6—C16	1.291 (2)	C13—H13	0.9300
O4—C11	1.375 (2)	C28—H28	0.9300
O4—C12	1.421 (3)	C17—C18	1.396 (3)
O5—C16	1.226 (2)	C3—H3	0.9300
N2—C23	1.295 (2)	C3—C4	1.377 (3)
N2—C22	1.433 (2)	C18—H18	0.9300
N1—C7	1.429 (2)	C18—C19	1.378 (3)
N1—C8	1.295 (2)	C21—H21	0.9300
O8—C26	1.375 (2)	C21—C20	1.389 (3)
O8—C27	1.419 (3)	C19—H19	0.9300
C1—C2	1.503 (3)	C19—C20	1.383 (3)
C15—C9	1.406 (2)	C5—H5	0.9300
C15—C14	1.398 (2)	C5—C4	1.385 (3)
C7—C2	1.403 (2)	C25—H25	0.9300
C7—C6	1.396 (3)	C20—H20	0.9300
C30—C29	1.403 (3)	C4—H4	0.9300
C30—C24	1.412 (3)	C12—H12A	0.9600
C10—H10	0.9300	C12—H12B	0.9600
C10—C9	1.412 (2)	C12—H12C	0.9600
C10—C11	1.370 (3)	C27—H27A	0.9600
C23—H23	0.9300	C27—H27B	0.9600
C23—C24	1.437 (3)	C27—H27C	0.9600
C2—C3	1.390 (3)		
			
O7—Cu1—Cu2	40.37 (3)	O6—C16—C17	117.57 (16)
O3—Cu1—Cu2	39.35 (4)	O5—C16—O6	123.24 (18)
O3—Cu1—O7	79.07 (5)	O5—C16—C17	119.07 (17)
O3—Cu1—N1	93.19 (6)	C17—C22—N2	119.80 (16)
O2—Cu1—Cu2	134.83 (4)	C21—C22—N2	120.50 (17)
O2—Cu1—O7	98.20 (5)	C21—C22—C17	119.66 (17)
O2—Cu1—O3	166.96 (6)	C7—C6—H6	119.5
O2—Cu1—N1	93.64 (6)	C5—C6—C7	121.07 (18)
N1—Cu1—Cu2	131.52 (4)	C5—C6—H6	119.5
N1—Cu1—O7	158.21 (6)	C15—C9—C10	120.11 (16)
O7—Cu2—Cu1	41.44 (4)	C15—C9—C8	124.40 (16)
O7—Cu2—O1^i^	93.92 (5)	C10—C9—C8	115.47 (16)
O3—Cu2—Cu1	38.01 (4)	C30—C24—C23	125.75 (16)
O3—Cu2—O7	78.81 (5)	C30—C24—C25	119.47 (17)
O3—Cu2—O1^i^	85.67 (5)	C25—C24—C23	114.76 (16)
O1^i^—Cu2—Cu1	84.04 (3)	O4—C11—C13	116.30 (16)
O6—Cu2—Cu1	128.39 (4)	C10—C11—O4	124.59 (17)
O6—Cu2—O7	143.58 (6)	C10—C11—C13	119.10 (17)
O6—Cu2—O3	95.61 (5)	O8—C26—C28	115.25 (17)
O6—Cu2—O1^i^	121.77 (6)	C25—C26—O8	125.38 (18)
O6—Cu2—N2	94.18 (6)	C25—C26—C28	119.37 (17)
N2—Cu2—Cu1	134.27 (4)	C15—C14—H14	119.5
N2—Cu2—O7	95.11 (6)	C13—C14—C15	120.90 (17)
N2—Cu2—O3	169.71 (6)	C13—C14—H14	119.5
N2—Cu2—O1^i^	86.48 (6)	C11—C13—H13	119.5
Cu2—O7—Cu1	98.18 (5)	C14—C13—C11	121.08 (17)
C30—O7—Cu1	132.32 (11)	C14—C13—H13	119.5
C30—O7—Cu2	125.98 (11)	C29—C28—C26	120.69 (18)
Cu1—O3—Cu2	102.64 (6)	C29—C28—H28	119.7
C15—O3—Cu1	124.31 (11)	C26—C28—H28	119.7
C15—O3—Cu2	129.03 (11)	C22—C17—C16	124.12 (16)
C1—O2—Cu1	129.79 (12)	C18—C17—C16	117.36 (17)
C1—O1—Cu2^i^	113.00 (12)	C18—C17—C22	118.52 (18)
C16—O6—Cu2	126.44 (12)	C2—C3—H3	118.7
C11—O4—C12	116.00 (15)	C4—C3—C2	122.61 (19)
C23—N2—Cu2	123.84 (13)	C4—C3—H3	118.7
C23—N2—C22	118.75 (15)	C17—C18—H18	119.1
C22—N2—Cu2	116.89 (11)	C19—C18—C17	121.76 (19)
C7—N1—Cu1	120.52 (11)	C19—C18—H18	119.1
C8—N1—Cu1	122.04 (12)	C22—C21—H21	119.7
C8—N1—C7	117.43 (15)	C20—C21—C22	120.51 (19)
C26—O8—C27	116.52 (16)	C20—C21—H21	119.7
O2—C1—C2	120.14 (15)	C18—C19—H19	120.3
O1—C1—O2	121.74 (17)	C18—C19—C20	119.31 (19)
O1—C1—C2	118.12 (16)	C20—C19—H19	120.3
O3—C15—C9	121.09 (15)	C6—C5—H5	119.9
O3—C15—C14	120.93 (16)	C6—C5—C4	120.21 (19)
C14—C15—C9	117.97 (16)	C4—C5—H5	119.9
C2—C7—N1	120.78 (16)	C24—C25—H25	119.5
C6—C7—N1	120.26 (16)	C26—C25—C24	120.96 (18)
C6—C7—C2	118.96 (16)	C26—C25—H25	119.5
O7—C30—C29	120.21 (16)	C21—C20—H20	119.9
O7—C30—C24	121.92 (16)	C19—C20—C21	120.2 (2)
C29—C30—C24	117.86 (16)	C19—C20—H20	119.9
C9—C10—H10	119.7	C3—C4—C5	118.65 (18)
C11—C10—H10	119.7	C3—C4—H4	120.7
C11—C10—C9	120.61 (17)	C5—C4—H4	120.7
N2—C23—H23	116.4	O4—C12—H12A	109.5
N2—C23—C24	127.25 (16)	O4—C12—H12B	109.5
C24—C23—H23	116.4	O4—C12—H12C	109.5
C7—C2—C1	125.76 (16)	H12A—C12—H12B	109.5
C3—C2—C1	115.76 (16)	H12A—C12—H12C	109.5
C3—C2—C7	118.47 (17)	H12B—C12—H12C	109.5
C30—C29—H29	119.2	O8—C27—H27A	109.5
C28—C29—C30	121.65 (18)	O8—C27—H27B	109.5
C28—C29—H29	119.2	O8—C27—H27C	109.5
N1—C8—H8	116.7	H27A—C27—H27B	109.5
N1—C8—C9	126.52 (16)	H27A—C27—H27C	109.5
C9—C8—H8	116.7	H27B—C27—H27C	109.5

Symmetry code: (i) −*x*+1, −*y*+1, −*z*+1.

### Bis{µ4-(E)-2-[(5-methoxy-2-oxidobenzylidene)amino]benzoato}bis{µ2-(E)-2-[(5-methoxy-2-oxidobenzylidene)amino]benzoato}tetracopper(II) Hydrogen-bond geometry (Å, º)

**Table:**

*D*—H···*A*	*D*—H	H···*A*	*D*···*A*	*D*—H···*A*
C14—H14···O6	0.93	2.55	3.112 (3)	119
C29—H29···O2	0.93	2.28	2.945 (2)	128
C23—H23···O5^ii^	0.93	2.58	3.432 (2)	153
C27—H27*A*···O5^iii^	0.96	2.63	3.567 (3)	165

Symmetry codes: (ii) −*x*+1, −*y*, −*z*; (iii) *x*−1, *y*, *z*.
